# Predicting comorbidities in psoriasis using TCM constitution and multimodal data: a prospective cohort study protocol

**DOI:** 10.3389/fmed.2026.1855767

**Published:** 2026-07-21

**Authors:** Jing Hu, Di Long, Junchen Chen, Meijunzi Luo, Rong Zhou, Xuanchun Zhang, Ziwan Lei, Wenyu Deng, Yehong Kuang, Haizhen Wang

**Affiliations:** 1 The Second Affiliated Hospital of Hunan University of Chinese Medicine, Changsha, China; 2 Xiangya Hospital Central South University, Changsha, China

**Keywords:** comorbidities, prospective cohort study, psoriasis, TCM constitution, TCM multimodal information

## Abstract

**Background:**

Psoriasis is a chronic inflammatory skin disease associated with multiple systemic comorbidities. Patients exhibit considerable individual variability in response to systemic therapies, making treatment outcomes and adverse reactions difficult to predict. Traditional Chinese Medicine (TCM) constitution theory may explain this variability, but prospective validation is lacking. This study primarily aims to evaluate the predictive value of TCM constitution types and multimodal clinical data for comorbidity risk in patients with psoriasis. As a secondary objective, it will also explore the association between TCM constitution and systemic treatment effectiveness and adverse events.

**Methods:**

This is a multicenter, prospective, observational cohort enrolling 1000 adults (≥18 years) with psoriasis who are scheduled to initiate systemic therapy. Over a 52-week period, we will collect comprehensive data on TCM constitution types, syndrome patterns, and multimodal clinical data. All participants will be followed to monitor comorbidity progression and, secondarily, to assess the effectiveness of systemic therapies and record adverse events.

**Discussion:**

By leveraging a digitized platform for TCM and clinical data collection, this prospective study aims to validate the relationship between TCM constitution types and psoriasis comorbidities and, secondarily, treatment responses. The ultimate objective is to build integrated predictive models for comorbidity risk as the primary outcome and treatment outcomes as secondary endpoints, combining TCM-based features with modern clinical indicators to advance personalized medicine in psoriasis.

**Ethics and dissemination:**

The study protocol was approved by the Medical Ethics Review Committee of The Second Affiliated Hospital of Hunan University (ID: 2025-KY-036-01) and the Medical Ethics Review Committee of Xiangya Hospital, Central South University (ID: 2025122317). Study results will be published in peer-reviewed journals and presented at national and international conferences. In addition, lay summaries of the findings will be made available to participants and the public via the hospitals’ websites and other public forums.

**Clinical trial registration:**

https://www.chictr.org.cn/searchprojEN.html, identifier [ITMCTR2025002518].

## 1 Introduction

### 1.1 Background and rationale

Psoriasis is a chronic inflammatory dermatological condition mediated by immune dysregulation, primarily characterized by the presence of erythematous plaques with silvery scales (1). Its global prevalence is approximately 2%–4% (2), and its onset is closely associated with genetic, immunological, and environmental factors (3). Psoriasis is frequently accompanied by a range of comorbidities, including psoriatic arthritis, cardiovascular disease, metabolic syndrome, inflammatory bowel disease, and anxiety (4–6). The 2023 Chinese Guidelines for Psoriasis define comorbidities as systemic diseases that frequently co-occur with psoriasis beyond its cutaneous manifestations. These associated conditions include, but are not limited to, cardiovascular diseases, metabolic disorders, hepatic and renal impairments, autoimmune diseases, and psychiatric conditions. Currently, these diseases, which exhibit a significant association with psoriasis, are termed psoriasis comorbidities (5, 7).

In recent years, the use of biologics has significantly improved the prognosis for patients with moderate-to-severe psoriasis. However, clinical practice reveals substantial individual variation in treatment response: while some patients achieve sustained remission, others exhibit suboptimal responses, experience treatment failure, or develop adverse effects (8). The mechanisms underlying this discrepancy remain incompletely elucidated, and there is currently a lack of effective predictive tools to assess individual patient treatment outcomes, comorbidity risk, and long-term prognosis.

Traditional Chinese medicine theory emphasizes an integrated holistic approach and individualized differentiation, offering a unique perspective for understanding these variations. Central to this is the concept of TCM constitution, which posits that constitutional traits—such as Balanced Constitution, Qi Deficiency Constitution, Damp-Heat Constitution or Yang Deficiency Constitution—determine an individual’s susceptibility to disease, disease progression, and response to treatment (9). Different constitutional types may influence clinical manifestations of psoriasis (10), comorbidity patterns, and the effectiveness of biologic therapies by modulating immune status, inflammatory levels, and metabolic pathways. Chinese medicine diagnosis relies on the multimodal information obtained through “inspection, auscultation and olfaction, inquiry, and palpation” encompassing visual data such as tongue and facial features, and skin lesion morphology, as well as pulse signals and clinical symptoms and signs. These multimodal features contain extensive pattern differentiation information and serve as crucial diagnostic criteria for constitution and syndrome pattern identification.

Advances in artificial intelligence, particularly the maturity of deep learning in image recognition and predictive modeling (11), have created unprecedented opportunities for the systematic analysis of multimodal imaging data in TCM. By digitizing images of the tongue, face, and skin lesions and integrating them with clinical information, this approach offers the potential to establish an objective and quantifiable system of TCM pattern characteristics. The application of these features in large-scale, prospective cohort studies could not only validate the associations between TCM constitution, psoriasis comorbidities, and treatment effectiveness but also uncover novel predictive indicators.

Therefore, this study proposes to conduct a prospective cohort study of patients with psoriasis, systematically collecting data on TCM constitutional types, syndrome patterns, and multimodal imaging—including tongue features, facial features, and skin lesion images—in psoriasis patients. The study will include long-term follow-up to evaluate the short-term and long-term effectiveness of systemic therapies, monitor adverse effects, and observe the evolution of comorbidities. The aim is to investigate how different TCM constitutional types and patterns influence treatment outcomes, and to further integrate modern clinical indicators with digital TCM syndrome differentiation features. This will facilitate the development of a comprehensive predictive model capable of forecasting the risk of psoriasis-related comorbidities, treatment effectiveness, adverse reactions, and disease progression. The research findings are expected to advance precision treatment for psoriasis, promote the integration of TCM syndrome differentiation with modern therapeutic approaches, and provide a scientific basis for early clinical intervention and personalized treatment strategies, ultimately improving overall diagnostic and therapeutic levels for psoriasis.

## 2 Objectives

### 2.1 Primary objectives

To evaluate the predictive value of TCM constitution types and multimodal clinical data for the risk of developing new comorbidities in patients with psoriasis undergoing systemic therapy.

### 2.2 Secondary (exploratory) objectives

The following objectives are designated as secondary and hypothesis-generating: (1) To explore whether TCM constitution types modify systemic treatment effectiveness, as measured by PASI 75/90/100 response rates, within treatment-stratified subgroups. (2) To explore the association between TCM constitution types and the occurrence of treatment-emergent adverse events across different systemic therapy classes.

## 3 Methods and analysis

### 3.1 Study design

This study is a multicenter, prospective, observational cohort study conducted according to standard clinical management protocols, which exclude off-label or non-guideline-recommended pharmacotherapies. Initiated in December 2025, the study is currently recruiting participants and aims to enroll 1,000 eligible participants between December 2025 and December 2027. The design comprises two phases: a cross-sectional baseline assessment and a subsequent 52-week longitudinal follow-up.

**FIGURE 1:**
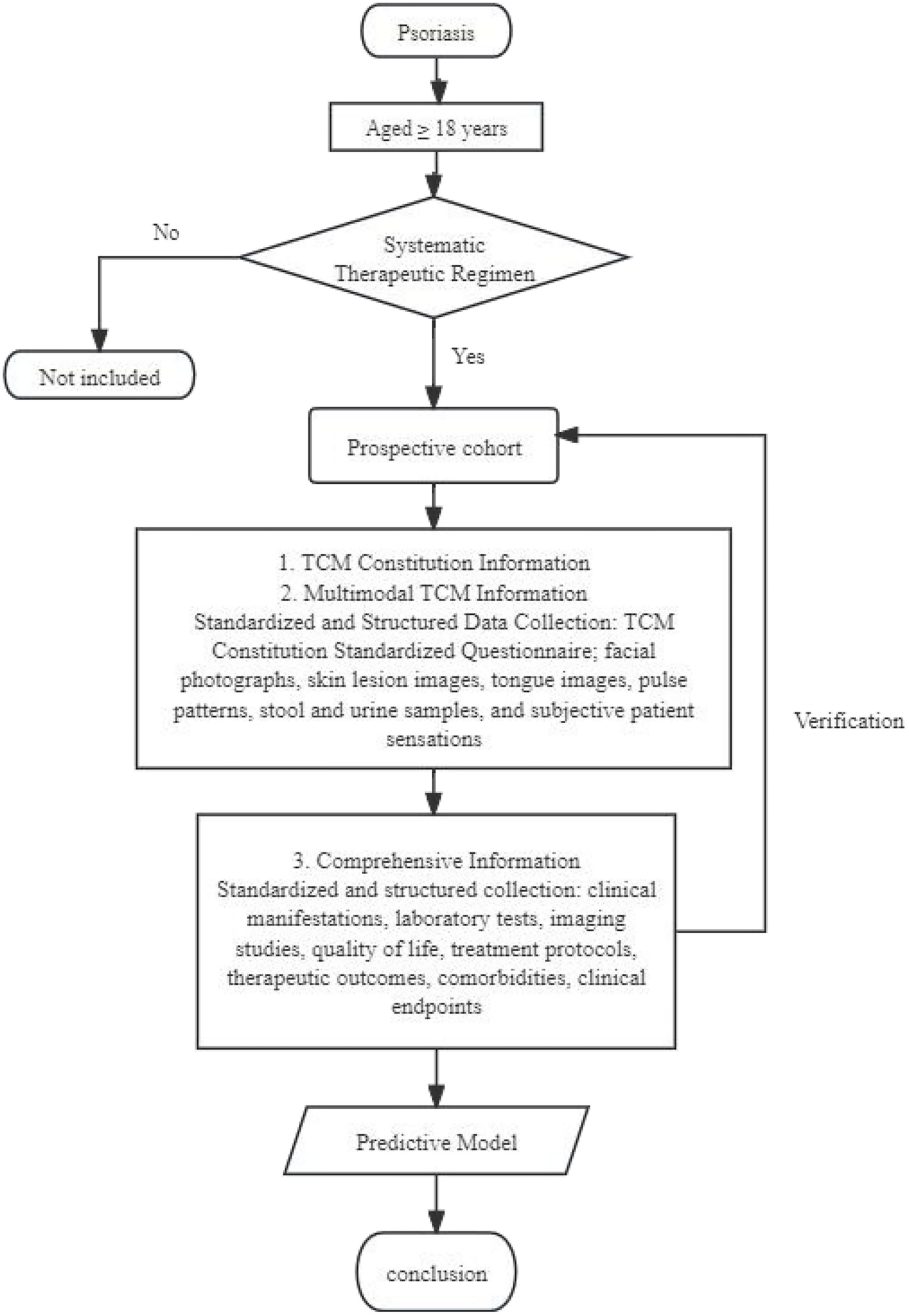
Technology roadmap.

This manuscript adheres to the guidelines set by the Strengthening the Reporting of Observational Studies in Epidemiology (STROBE) statement (12). The STROBE checklist (Supplementary Additional File 1) and SPIRIT checklist (Supplementary Additional File 2 and Figure 1) are provided.

### 3.2 Study settings and participants

This study is initiated and led by the Second Affiliated Hospital of Hunan University of Chinese Medicine, with plans to include two centers (the Second Affiliated Hospital of Hunan University of Chinese Medicine and Xiangya Hospital, Central South University). It is expected that complete 52-week follow-up data will be obtained from 1,000 patients.

### 3.3 Eligibility criteria

#### 3.3.1 Inclusion and exclusion criteria

Inclusion criteria were as follows: (1) patients diagnosed with psoriasis according to the 2023 Chinese Psoriasis Diagnosis and Treatment Guidelines; (2) aged ≥ 18 years, male or female; (3) scheduled to receive systemic therapy, including traditional systemic agents, biologics, and small-molecule targeted drugs; and (4) fully informed about the study and willing to comply with the study procedures. Exclusion criteria were as follows: (1) patients with other concomitant immune-mediated inflammatory diseases, including but not limited to rheumatoid arthritis, Crohn’s disease, and systemic lupus erythematosus; (2) patients lacking complete treatment and follow-up data; (3) patients with active malignant tumors; (4) patients with ongoing severe infections; (5) patients with active tuberculosis; and (6) pregnant women, those planning a pregnancy, or lactating women.

#### 3.3.2 Withdrawal criteria

Participants will be withdrawn if they meet specific discontinuation criteria, including major organ dysfunction, serious adverse events, poor compliance, disease progression, or any condition necessitating cessation of the study treatment. Participants may also voluntarily withdraw from the study for reasons including inadequate therapeutic response, intolerance to adverse effects, pursuit of alternative treatments, or withdrawal of consent for any reason.

#### 3.3.3 Discontinuation criteria

Patients who fail to complete the full schedule of visits due to adverse events, serious adverse events, loss to follow-up, lack of effectiveness, voluntary withdrawal of informed consent, or other reasons will be classified as discontinued.

#### 3.3.4 Study termination criteria

The criteria for suspension or termination of this study include the following: (1) medical or ethical grounds, where continuation is deemed inappropriate due to an unfavorable risk–benefit ratio, widespread or unanticipated serious adverse reactions, quality issues with the investigational product, or substantial safety concerns; (2) scientific grounds, including major protocol errors or significant deviations that prevent reliable evaluation of effectiveness or safety; (3) administrative reasons, such as changes in funding, management, or policy, provided patient safety is protected; and (4) regulatory or ethical directive, where termination is mandated by the National Medical Products Administration, the ethics committee, investigators, or the sponsor in accordance with Good Clinical Practice (GCP) and local regulations.

### 3.4 Data collection

#### 3.4.1 Baseline and follow-up data collection

At baseline, after obtaining informed consent and confirming eligibility, the following data will be collected from all participants: demographic characteristics (age, sex, education level, occupation, marital status, income, health insurance status) and anthropometric measurements (height, weight, waist circumference, hip circumference), from which body mass index (BMI) will be calculated; lifestyle information (smoking status, alcohol consumption); psoriasis clinical features (age at onset, disease duration, family history) and disease severity assessed by PASI, BSA, and PGA scores; patient-reported outcomes including the Dermatology Life Quality Index (DLQI) and Numerical Rating Scales (NRS, 0–10) for pruritus, pain, irritability, dry mouth, and fatigue; baseline comorbidities (psoriatic arthritis, hypertension, diabetes mellitus, and other conditions as defined in section “3.8.1 Primary endpoint”); previous treatment history (duration, adherence, regimens); concomitant medications; and physical examination findings. Auxiliary laboratory tests (complete blood count, urinalysis, stool examination, blood biochemistry, ESR, CRP, infectious disease screening, chest CT) will be performed according to hospital protocols. During the 52-week follow-up, disease severity (PASI, BSA, PGA), patient-reported outcomes (DLQI, NRS), concomitant medications, adverse events, and treatment adherence will be recorded at each visit (Weeks 4, 12, 24, and 52). All PASI, BSA, and PGA assessments will be performed by trained dermatologists blinded to the patient’s TCM constitution type and multimodal TCM data; patient-reported outcomes are collected directly from participants, independent of clinician assessment. TCM constitution will be re-assessed at Weeks 12 and 52 and treated as a time-varying covariate in statistical analyses. The complete schedule of enrolment, data collection, and observations is presented in Table 1.

**TABLE 1:** The schedule of enrolment, data collection and observations.

Observation/procedure	Screening (V1, 0 week)	Visit 2 (V2, ±4 weeks)	Visit 3 (V3, ±12 weeks)	Visit 4 (V4, ±24 weeks)	Visit 5 (V5, ±52 weeks)
Demographic information^a^	×	–	–	–	–
Lifestyle factors^b^	×	–	–	–	–
Disease characteristics^c^	×	–	–	–	–
Complications	×	×	×	×	×
Past treatment history^d^	×	–	–	–	–
Current treatment regimen^e^	×	×	×	×	×
Auxiliary examinations may include^f^	×	–	×	×	×
Disease severity scoring^g^	×	×	×	×	×
Patient-reported outcomes^h^	×	×	×	×	×
Adverse events	×	×	×	×	×
TCM constitution information^i^	×	–	×	–	×
Multimodal TCM information^j^	×	–	×	–	×

^a^Demographic information: age, gender, educational level, occupation, marital status, income, health insurance status, height, weight, waist circumference, hip circumference.

^b^Lifestyle factors: smoking, alcohol consumption.

^c^Disease characteristics: age at onset, disease duration, lesion sites, family history.

^d^Past treatment history: duration, adherence, treatment regimens and durations.

^e^Current treatment regimen: dosage, adherence, treatment plan.

^f^Auxiliary examinations may include: complete blood count, urinalysis, stool analysis, blood biochemistry, fasting blood glucose, lipid profile, coronary artery disease risk factors, erythrocyte sedimentation rate (ESR), C-reactive protein (CRP), infectious disease screening, chest CT (specific auxiliary tests to follow local hospital diagnostic protocols).

^g^Disease severity scoring: PASI score, BSA score, PGA score.

^h^Patient-reported outcomes: DLQI score, PtGA score.

^i^TCM constitution information: the nine TCM constitutions—Balanced Constitution, Qi Deficiency Constitution, Yang Deficiency Constitution, Yin Deficiency Constitution, Phlegm-Dampness Constitution, Damp-Heat Constitution, Blood Stasis Constitution, Qi Stagnation Constitution, and Inherited Special Constitution. Assessed through standardized questionnaires completed by patients with researcher assistance, with constitution scores calculated for evaluation.

^j^Multimodal TCM information: frontal facial photograph (standard colorimetric card), tongue image (standard colorimetric card), skin lesion photograph (standard colorimetric card), pulse diagnosis, stool frequency, stool characteristics, urine color, subjective symptoms NRS (itching NRS, pain NRS, irritability/restlessness NRS, dry mouth and tongue NRS, fatigue NRS).

All PASI, BSA, and PGA assessments will be performed by trained dermatologists who are blinded to the patient’s TCM constitution type and other multimodal TCM data. TCM constitution and related data are stored in a separate, access-restricted database that is not accessible to the outcome assessors until after the final 52-week follow-up and database lock.

#### 3.4.2 Multimodal TCM data and constitution assessment

Multimodal TCM data are collected following national standards (13–16) with uniform equipment across centers (Hi-Face22 for facial and tongue imaging; Hi-W-7 for pulse acquisition; standardized camera with 24-color calibration card for lesion photography). The instruments automatically extract: chromaticity values (CIELAB L*, a*, b*), skin texture indices, tongue body parameters (tooth-mark, crack, plumpness indices), and tongue coating characteristics (thickness, color, moisture). Auscultation and olfaction findings are recorded as categorical variables. Inquiry data—including stool frequency, urine color, and symptom NRS scores—are collected via structured questionnaire. Pulse features automatically extracted include time-domain parameters (pulse rate, width *w*, area S, S2), frequency-domain parameters (spectral energy ratios), and categorical assessments at bilateral cun, guan, and chi positions. A quality assurance framework minimizes inter-center heterogeneity (see Quality Control).

Traditional Chinese Medicine constitution is assessed using Hi-Face22. Before 1 April 2026, classification follows ZYYXH/T157-2009 (17); thereafter, GB/T 46939-2025 (18). Both use identical nine-type classification and transformed score formula: [(Raw Score–Number of Items)/(Number of Items × 4)] × 100. Constitution types—Balanced, Qi Deficiency, Yang Deficiency, Yin Deficiency, Phlegm-Dampness, Damp-Heat, Blood Stasis, Qi Stagnation, and Intrinsic Special Constitution—are recorded at baseline and Weeks 12 and 52.

#### 3.4.3 Data processing and variable specification

All raw data are converted into analyzable variables through three pipelines: (1) automated extraction—constitution scores, imaging features, and pulse parameters are computed by Hi-Face22 and Hi-W-7; (2) standardized clinician assessment—PASI, BSA, and PGA are rated by blinded dermatologists; (3) structured recording—patient-reported outcomes, auscultation findings, and demographics are captured via structured case report forms. The mapping of raw data to analyzable variables is summarized in Table 2.

**TABLE 2:** Summary of feature extraction by modality.

Modality	Variables/features extracted	Extraction method
TCM constitution	Primary constitution type (categorical); Transformed scores for nine constitution types	Automated (Hi-Face22 instrument)
Facial image	Chromaticity values (L*, a*, b*); Skin texture indices	Automated (Hi-Face22 built-in algorithm)
Tongue image	Tongue body color (hue, saturation); Shape parameters (tooth-mark, crack, plumpness indices); Coating characteristics (thickness grade, color, moisture)	Automated (Hi-Face22 built-in algorithm)
Skin lesions	PASI, BSA, PGA scores	Manual (blinded dermatologist-rated)
Pulse waveform	Time-domain parameters (pulse rate, width *w*, area *S*, *S2*); Frequency-domain parameters; Categorical assessments (position, rate, form, strength)	Automated (Hi-W-7 pulse device)
Patient-reported outcomes	DLQI total score; NRS scores (pruritus, pain, irritability, dry mouth, fatigue)	Manual (patient-reported)
Auscultation and olfaction	Vocal quality (normal/hoarse/low/high); Odors (presence/absence)	Manual (physician-rated)
Demographics and comorbidities	Age, gender, BMI, smoking, alcohol, psoriatic arthritis, hypertension, diabetes, etc.	Manual (structured CRF)

Hi-Face22, unified TCM constitution identification instrument with integrated imaging module, centrally procured across all centers; Hi-W-7, certified intelligent pulse diagnosis device, uniformly deployed across all centers; Blinding, all clinician-rated assessments (PASI, BSA, PGA) are performed by dermatologists blinded to TCM constitution data. For details of quality control (daily 24-color card calibration, quarterly pulse phantom verification), see the “Standardized Multi-Center Acquisition and Quality Control” subsection.

Variables are organized into three tiers: (a) core predictor—TCM constitution type (nine-level categorical, entered as the stratification factor in the primary stratified Cox model; recoded as eight dummy variables with Balanced Constitution as reference in supplementary analyses); (b) supplementary covariates—multimodal TCM features (tongue/facial chromaticity, tongue shape and coating parameters, pulse time-domain and frequency-domain parameters, NRS scores), clinical severity (PASI, BSA, PGA), DLQI, age, sex, and baseline comorbidity count—all entering the primary model as covariates; (c) exploratory—auscultation/olfaction ratings, screened but not pre-specified for the primary model.

#### 3.4.4 Key variable specification

Stratification factor: TCM constitution type (nine-level categorical).

Primary model covariates: Age (continuous, years); sex (binary); BMI (continuous, kg/m^2^); disease duration (continuous, years); baseline PASI (continuous, 0–72); baseline comorbidity count (continuous); treatment type (time-varying categorical: traditional systemic agents, biologics, small-molecule targeted drugs).

Primary endpoint: Time to first target comorbidity (see section “3.8.1 Primary endpoint”); censored at last follow-up if event-free.

Secondary endpoints: PASI 75/90/100 response (binary, calculated as [(baseline PASI–week *t* PASI)/baseline PASI] × 100%); PASI, BSA, DLQI as continuous; adverse events graded by CTCAE v5.0, coded by MedDRA.

Multimodal feature selection: Candidate features from tongue, facial, pulse, and lesion imaging are screened via LASSO with 10-fold cross-validation; only non-zero coefficient features are retained. Center will be included as a random effect, with ComBat harmonization applied to image-derived features in sensitivity analyses.

### 3.5 Data management

#### 3.5.1 Data collection and entry

Researchers should base their data entry on original observation records, ensuring the completeness, accuracy, and timeliness of case report form data. Monitors are responsible for verifying that the study adheres to the protocol, confirming that the report forms are consistent with the original data, and promptly requesting corrections for any errors. Corrections must be signed and dated; the use of erasers or correction fluid is prohibited. Report forms reviewed by monitors should be submitted to the data manager in a timely manner, with the transfer process documented and properly stored. Before the initial submission of the first report form, the data manager should establish a comprehensive and secure database and conduct preliminary reviews of each report form. Double data entry by two independent operators should be employed, with software comparison to identify discrepancies, ensuring data consistency.

#### 3.5.2 Data verification and cleaning

The data administrator shall develop procedures in accordance with the verification plan to identify potential errors or queries. Any issues detected must be promptly notified to the data monitor, with the investigator required to provide a response. Queries and resolutions shall be documented using the Query Form, including data supplementation and re-verification. All records and modification outcomes must be meticulously preserved.

#### 3.5.3 Generation of key new variables, medical coding, and de-identification

Critical clinical variables will be organized, new variables will be created, and medical coding will be applied using international medical dictionaries (e.g., MedDRA for adverse events, WHO Drug Dictionary for concomitant medications). Personally identifiable information will be removed, personal identification data stored separately, and only a unique study subject identifier retained in the database to ensure privacy and data security. All derived variables, including TCM constitution transformed scores and multimodal imaging features automatically extracted by the Hi-Face22 and Hi-W-7 devices, will be generated programmatically using prespecified algorithms, with full documentation of variable names, labels, and derivation rules stored in the data dictionary.

#### 3.5.4 Data verification and locking

After data cleaning, the research team cross-checks the database for dropout cases, effectiveness, and safety data through blinded review. Once verified for accuracy, the database is locked to prevent any modifications, ensuring data integrity. Subsequently, the locked database is handed over to statisticians for analysis. The blinding protocol ensures that the statisticians performing the primary analyses remain blinded to group allocation until after the final statistical analysis plan is executed.

### 3.6 Quality control

An electronic data collection system will be established. All data will be entered by two independent researchers and subjected to consistency verification. A Standard Operating Procedure for Quality Control will be developed and adhered to, with regular data audits conducted.

#### 3.6.1 Standardized multi-center acquisition and quality control for multimodal data

To minimize inter-center and inter-visit heterogeneity in the multi-center, 52-week follow-up setting, the following quality assurance measures are implemented across all participating sites.

(1) Equipment uniformity and personnel training

All centers use centrally procured identical devices: Hi-Face22 TCM constitution identification instrument (with integrated imaging module for facial and tongue image capture) and Hi-W-7 pulse diagnosis device. All operators complete centralized training and certification according to a standardized SOP manual prior to study initiation.

(2) Image quality control

Facial and tongue images are acquired by the Hi-Face22 inside a light-shielded enclosure with controlled LED illumination. Before each daily acquisition session, the operator photographs a 24-color calibration card compliant with ISO/TS 20498-3:2020. The system automatically calculates the mean ΔE*ab across all patches; acquisition proceeds only if mean ΔE*ab ≤ 5, consistent with the ISO/TS 20498-3:2020 tolerance specification for computerized tongue image analysis systems. All calibration checks are automatically logged with date/time stamps and center codes for complete traceability. For skin lesion photographs taken with the standardized professional camera, a 24-color calibration card is included in every image for post-hoc color correction.

(3) Pulse waveform calibration

Each Hi-W-7 pulse diagnosis device undergoes factory calibration traceable to a reference pressure waveform simulator prior to deployment. At each power-on, the device performs automated self-checks (zero-offset correction and pressure-sensor baseline verification); acquisition is only permitted when all self-check parameters are within the manufacturer-specified range. Additionally, quarterly phantom verification is conducted at each center; deviation of key parameters (pulse width *w*, pulse area S) exceeding 5% triggers device recalibration. All calibration logs are retained in the electronic data capture system.

(4) Blinded assessment

Traditional Chinese Medicine constitution assessment, clinical severity scoring (PASI, BSA, PGA), and patient-reported outcomes are collected by separate investigators who are blinded to each other’s assessments. TCM constitution and related multimodal data are stored in a separate, access-restricted database not accessible to outcome assessors until after database lock. This blinding protocol extends to statisticians performing the primary analyses, minimizing observer and analytical bias.

(5) Inter-center and inter-visit consistency

All study procedures—including patient positioning, image capture protocols, and device operation—follow a unified SOP. multimodal data acquisition conditions (lighting, camera settings, pulse device parameters) are standardized across all centers and visits, ensuring reproducibility throughout the 52-week follow-up period.

### 3.7 Research grouping

This is a prospective cohort study without pre-defined comparator groups at enrollment. Grouping will be performed during the analysis phase according to the study objectives.

#### 3.7.1 Core stratification for the primary objective

For the primary objective of predicting comorbidity risk, all enrolled patients will be classified at baseline according to the Classification and Determination of TCM Constitutions standard into nine constitutional types: Balanced Constitution, Qi Deficiency Constitution, Yang Deficiency Constitution, Yin Deficiency Constitution, Phlegm-Dampness Constitution, Damp-Heat Constitution, Blood Stasis Constitution, Qi Stagnation Constitution, and Intrinsic Special Constitution. TCM constitution type serves as the single core stratification variable for the primary comorbidity risk analysis. During follow-up, patients will be grouped by the occurrence of new comorbidities: event group—patients newly diagnosed with any target comorbidity during the 52-week follow-up; non-event group—patients who remain free of target comorbidities through the end of follow-up.

#### 3.7.2 Secondary (exploratory) groupings

For exploratory objectives, patients will be stratified by systemic treatment class, with further subgrouping by TCM constitution to examine potential effect modification. These groupings are hypothesis-generating and are not part of the primary analytical framework.

#### 3.7.3 Data-driven subtype exploration

Unsupervised learning methods will be applied to integrated multimodal clinical data to identify potential intrinsic disease subtypes and explore their associations with clinical outcome trajectories.

### 3.8 Outcome measures

#### 3.8.1 Primary endpoint

The primary endpoint is time from enrollment to the first documented diagnosis of any target comorbidity during the 52-week follow-up. Target comorbidities include psoriatic arthritis, cardiovascular diseases (hypertension, coronary artery disease, myocardial infarction), metabolic syndrome, type 2 diabetes, non-alcoholic fatty liver disease, inflammatory bowel disease, and depression/anxiety disorders, all diagnosed according to standardized international criteria. Patients who do not develop any target comorbidity will be censored at their last study visit. The cumulative number and types of incident comorbidities will also be recorded. This endpoint directly corresponds to the primary objective and serves as the dependent variable for the primary stratified Cox regression analysis.

#### 3.8.2 Exploratory endpoints

The following endpoints serve secondary, hypothesis-generating objectives. They do not inform the sample size calculation, and analyses involving these endpoints will not be adjusted for multiplicity.

(1) Effectiveness endpoints

Proportion of patients achieving PASI 90 response at Weeks 4, 12, 24, and 52, as the primary effectiveness measure of interest; PASI 75 and PASI 100 response rates will also be reported. Absolute change from baseline in PASI, BSA, and DLQI scores at each follow-up visit.

(2) Safety endpoints

Incidence and severity of treatment-emergent adverse events (TEAEs), graded by CTCAE version 5.0 and coded by MedDRA.

Incidence of clinically significant laboratory abnormalities (≥Grade 2 CTCAE), with repeat testing until resolution or return to baseline.

Adverse events of special interest: serious infections, malignancies, major adverse cardiovascular events, hepatitis B reactivation, and active tuberculosis.

All adverse events will be documented in the CRF from informed consent through the last follow-up. For patients receiving multiple treatment courses, safety data will be analyzed per course.

#### 3.8.3 Data-driven subtypes (tertiary exploration)

Unsupervised learning methods (e.g., cluster analysis) will be applied to integrated multimodal clinical data to identify potential intrinsic disease subtypes and explore their associations with comorbidity incidence, treatment response, and adverse-event profiles. This analysis is purely hypothesis-generating.

### 3.9 Sample size calculation

According to the commonly used 10EPP rule in clinical prediction models—where each parameter requires at least 10 outcome events—each predictive variable should be associated with a minimum of 10 events (19). The primary prediction model includes approximately 10 candidate predictor variables: eight TCM constitution types (dummy variables, with Balanced Constitution as reference), plus age, sex, BMI, disease duration, baseline PASI, baseline comorbidity count, and treatment type (modeled as a time-varying covariate). Consequently, a minimum of 100 outcome events is required.

Based on a large-scale clinical characteristics analysis of Chinese psoriasis patients, the comorbidity prevalence was reported as 16.79% (20). This study conservatively estimates the incidence of new-onset comorbidities at approximately 20% over the 52-week follow-up. Accordingly, the theoretical minimum sample size is calculated as 100 ÷ 0.20 = 500 participants. Accounting for an estimated 15% rate of loss to follow-up and data missingness, the target sample size is set at 1,000 participants, which is expected to yield approximately 200 outcome events, providing a comfortable margin above the minimum requirement.

To address the risk of overfitting, LASSO regression with 10-fold cross-validation will be applied for data-driven variable selection, retaining only predictors with non-zero coefficients, and internal validation will be performed via bootstrap resampling (1,000 resamples) to obtain optimism-corrected estimates of model discrimination (Harrell’s C-index) and calibration. Missing data, if exceeding 5% for any variable, will be handled using multiple imputation by chained equations (MICE) with *m* = 20 imputed datasets, with complete-case analysis reported as a sensitivity analysis.

This sample size is comparable to published prediction model studies in psoriasis and is achievable within the planned 24-month recruitment period across two centers (21). The secondary, exploratory analyses of treatment effectiveness and adverse events are not factored into this sample size calculation.

### 3.10 Statistical analysis

All statistical analyses will be performed using R software (version 4.3.0; R Foundation for Statistical Computing, Vienna, Austria). Continuous variables will be assessed for normality using the Shapiro–Wilk test. Normally distributed data will be expressed as mean ± standard deviation (SD), and non-normally distributed data as median (interquartile range, IQR). Categorical variables will be summarized as frequencies (percentages). All statistical tests will be two-sided, and a *p*-value < 0.05 will be considered statistically significant unless otherwise specified.

#### 3.10.1 Descriptive analysis of baseline characteristics

Baseline demographic, clinical, and TCM constitution characteristics will be summarized for the entire cohort and further stratified by the nine TCM constitution types. Group comparisons across constitution types will be performed using one-way analysis of variance or the Kruskal–Wallis test for continuous variables, and the chi-squared test or Fisher’s exact test for categorical variables, as appropriate.

#### 3.10.2 Primary analysis: prediction of comorbidity risk

The primary endpoint is time from enrollment to the first diagnosis of any new comorbidity, with patients not developing an event censored at their last follow-up visit. A stratified Cox proportional hazards model will be constructed with the nine TCM constitution types as the stratification factor, allowing distinct baseline hazard functions for each constitution. The following pre-specified covariates will be entered as independent variables: age (continuous), sex (binary), body mass index (continuous), disease duration (continuous), baseline PASI score (continuous), baseline comorbidity count (continuous), and treatment type (modeled as a time-varying covariate: traditional systemic agents, biologics, small-molecule targeted drugs). LASSO regression with 10-fold cross-validation will be applied for variable selection, retaining predictors with non-zero coefficients. Model discrimination will be assessed using Harrell’s C-index, and calibration will be evaluated using calibration plots. Internal validation will be performed via bootstrap resampling (1,000 resamples) to obtain optimism-corrected performance estimates. Treatment-by-constitution interaction terms will be tested in a non-stratified Cox model, with *p* < 0.10 considered suggestive. The proportional hazards assumption will be tested using scaled Schoenfeld residuals. If any covariate has >5% missing data, multiple imputation by chained equations will be performed as a sensitivity analysis, with complete-case analysis reported for comparison.

#### 3.10.3 Secondary (exploratory) analyses

Secondary analyses of treatment effectiveness and adverse events are hypothesis-generating, will be conducted within treatment-stratified subgroups without multiplicity adjustment, and do not inform the sample size calculation.

### 3.11 Patient and public involvement

Patients and the public were not involved in the design, conduct, reporting, or dissemination plans of this research.

## 4 Discussion

Current research efforts in psoriasis have predominantly focused on identifying biomarkers predictive of treatment response, primarily within the domains of genomics and serological indicators (22, 23). Previous cross-sectional studies have reported associations between specific TCM constitution types—such as blood stasis and damp-heat types—and clinical manifestations of psoriasis (10). The present study addresses a critical gap by prospectively evaluating the predictive value of TCM constitution types and multimodal clinical data for comorbidity risk as the primary objective, while exploring treatment effectiveness and adverse events as secondary, hypothesis-generating objectives. This prioritized design ensures that the primary analysis remains focused and adequately powered, avoiding the dilution of statistical efficiency that can arise from multiple competing endpoints. Several methodological considerations inherent to the study design warrant explicit discussion.

First, this study explicitly prioritizes comorbidity risk prediction as the primary question, with a clearly defined primary endpoint of time to first target comorbidity. Treatment effectiveness and adverse events are designated as secondary, exploratory objectives and do not inform the sample size calculation. This hierarchy, consistently maintained from objectives through statistical analysis, directly addresses the concern that multiple outcome domains may reduce statistical efficiency and increase the risk of chance findings. All endpoints and their analysis plans have been pre-specified, and multiplicity adjustment is deliberately omitted for secondary analyses, with results to be interpreted as hypothesis-generating.

Second, substantial treatment heterogeneity exists among the three systemic therapy classes included in this cohort—traditional systemic agents, biologics, and small-molecule targeted drugs—which differ considerably in onset of action, effectiveness profiles, and adverse-event patterns. In the primary comorbidity prediction model, treatment type is handled as a time-varying covariate, thereby adjusting for its confounding effect while preserving the unified model structure. Treatment-stratified subgroup analyses and treatment-by-constitution interaction tests are reserved for secondary, exploratory objectives. However, these interaction analyses may be underpowered given the sample size, and their results will be interpreted with caution.

Third, the inclusion criteria no longer impose a minimum PASI threshold, as the primary objective concerns comorbidity risk across the full spectrum of patients requiring systemic therapy. While this enhances the generalizability of the comorbidity prediction model, it may introduce heterogeneity in baseline disease severity. To address this, baseline PASI, BSA, and DLQI are included as continuous covariates in all models, allowing the full range of severity to inform predictions without arbitrary dichotomization.

Fourth, the transformation of multimodal TCM data into analyzable variables follows three pre-specified pipelines—automated extraction, standardized clinician assessment, and structured recording—as detailed in section “3.4.4 Key variable specification.” Automated feature extraction by standardized instruments (Hi-Face22, Hi-W-7) ensures objectivity and reproducibility but constrains the feature space to those pre-specified by device algorithms, potentially omitting clinically salient patterns. LASSO regression with 10-fold cross-validation provides a data-driven mechanism to select the most informative features from the candidate pool, and bootstrap internal validation yields optimism-corrected performance estimates, mitigating overfitting risk. Despite these measures, the selected features remain bounded by the initial extraction algorithms, and future studies incorporating advanced deep learning-based feature extraction may capture additional predictive information.

Additional limitations warrant acknowledgment. As an observational study, residual confounding cannot be entirely eliminated despite statistical adjustment for known covariates. The enrolling centers are both tertiary hospitals in Hunan Province, which may limit geographic generalizability. Some comorbidities with long latency periods may not manifest within the 52-week follow-up window. Furthermore, external validation in independent, multi-ethnic cohorts will be essential before the prediction model can be recommended for clinical application.

Despite these limitations, if the study achieves its primary objective, the resulting model will provide clinicians with a novel quantitative tool for early identification of patients at elevated risk of comorbidity progression, enabling risk-stratified surveillance and preventive strategies. The exploratory findings on treatment effectiveness and safety may generate hypotheses for future studies examining whether TCM constitution-guided treatment selection improves outcomes. This work aims to translate the individualized TCM principle of “constitution-based prevention and treatment” into a practical clinical predictive instrument, fostering a deeper integration of traditional Chinese and Western medical approaches in the management of psoriasis comorbidities.
