# Supplementary material for: Predicting comorbidities in psoriasis using TCM constitution and multimodal data: a prospective cohort study protocol
Source: Front Med (Lausanne). 2026 Jul 21;13:1855767. doi: 10.3389/fmed.2026.1855767 (PMC13434845; doi:10.3389/fmed.2026.1855767)
Supplement: Supplementary file 1 [file Table_1.docx]

STROBE Statement—checklist of items that should be included in reports of observational studies

|  | Item No | Recommendation |
| --- | --- | --- |
| **Title and abstract** | 1 | (*a*) Indicate the study’s design with a commonly used term in the title or the abstract  **[Title and Abstract section]** |
|  |  | (*b*) Provide in the abstract an informative and balanced summary of what was done and what was found  **[Abstract section]** |
| Introduction | | |
| Background/rationale | 2 | Explain the scientific background and rationale for the investigation being reported  **[Background and rationale section]** |
| Objectives | 3 | State specific objectives, including any prespecified hypotheses  **[Study Objectives section]** |
| Methods | | |
| Study design | 4 | Present key elements of study design early in the paper  **[Study design section]** |
| Setting | 5 | Describe the setting, locations, and relevant dates, including periods of recruitment, exposure, follow-up, and data collection  **[Study settings and participants section]** |
| Participants | 6 | (*a*) *Cohort study*—Give the eligibility criteria, and the sources and methods of selection of participants. Describe methods of follow-up  *Case-control study*—Give the eligibility criteria, and the sources and methods of case ascertainment and control selection. Give the rationale for the choice of cases and controls  *Cross-sectional study*—Give the eligibility criteria, and the sources and methods of selection of participants |
|  |  | (*b*) *Cohort study*—For matched studies, give matching criteria and number of exposed and unexposed  *Case-control study*—For matched studies, give matching criteria and the number of controls per case  **[Eligibility criteria section]** |
| Variables | 7 | Clearly define all outcomes, exposures, predictors, potential confounders, and effect modifiers. Give diagnostic criteria, if applicable  **[Outcome Measures section]** |
| Data sources/ measurement | 8* | For each variable of interest, give sources of data and details of methods of assessment (measurement). Describe comparability of assessment methods if there is more than one group  **[Outcome Measures section]** |
| Bias | 9 | Describe any efforts to address potential sources of bias  **[Data management section，Quality control section]** |
| Study size | 10 | Explain how the study size was arrived at  **[Sample size calculation section]** |
| Quantitative variables | 11 | Explain how quantitative variables were handled in the analyses. If applicable, describe which groupings were chosen and why  **[Research Grouping section]** |
| Statistical methods | 12 | (*a*) Describe all statistical methods, including those used to control for confounding  **[Statistical analysis section]** |
|  |  | (*b*) Describe any methods used to examine subgroups and interactions  **[Statistical analysis section]** |
|  |  | (*c*) Explain how missing data were addressed  **[Not Applicable]** |
|  |  | (*d*) *Cohort study*—If applicable, explain how loss to follow-up was addressed  *Case-control study*—If applicable, explain how matching of cases and controls was addressed  *Cross-sectional study*—If applicable, describe analytical methods taking account of sampling strategy  **[Not Applicable]** |
|  |  | (*e*) Describe any sensitivity analyses  **[Not Applicable]** |

Continued on next page

| Results | | |
| --- | --- | --- |
| Participants | 13* | (a) Report numbers of individuals at each stage of study—eg numbers potentially eligible, examined for eligibility, confirmed eligible, included in the study, completing follow-up, and analysed  **[Not Applicable]** |
|  |  | (b) Give reasons for non-participation at each stage  **[Not Applicable]** |
|  |  | (c) Consider use of a flow diagram  **[Not Applicable]** |
| Descriptive data | 14* | (a) Give characteristics of study participants (eg demographic, clinical, social) and information on exposures and potential confounders  **[Data collection section]** |
|  |  | (b) Indicate number of participants with missing data for each variable of interest  **[Not Applicable]** |
|  |  | (c) *Cohort study*—Summarise follow-up time (eg, average and total amount)  **[Not Applicable]** |
| Outcome data | 15* | *Cohort study*—Report numbers of outcome events or summary measures over time  **[Not Applicable]** |
|  |  | *Case-control study—*Report numbers in each exposure category, or summary measures of exposure  **[Not Applicable]** |
|  |  | *Cross-sectional study—*Report numbers of outcome events or summary measures  **[Not Applicable]** |
| Main results | 16 | (*a*) Give unadjusted estimates and, if applicable, confounder-adjusted estimates and their precision (eg, 95% confidence interval). Make clear which confounders were adjusted for and why they were included  **[Not Applicable]** |
|  |  | (*b*) Report category boundaries when continuous variables were categorized  **[Not Applicable]** |
|  |  | (*c*) If relevant, consider translating estimates of relative risk into absolute risk for a meaningful time period  **[Not Applicable]** |
| Other analyses | 17 | Report other analyses done—eg analyses of subgroups and interactions, and sensitivity analyses  **[Not Applicable]** |
| Discussion | | |
| Key results | 18 | Summarise key results with reference to study objectives  **[Discussion section]** |
| Limitations | 19 | Discuss limitations of the study, taking into account sources of potential bias or imprecision. Discuss both direction and magnitude of any potential bias  **[Discussion section]** |
| Interpretation | 20 | Give a cautious overall interpretation of results considering objectives, limitations, multiplicity of analyses, results from similar studies, and other relevant evidence  **[Discussion section]** |
| Generalisability | 21 | Discuss the generalisability (external validity) of the study results  **[Discussion section]** |
|  | | |
| Funding | 22 | Give the source of funding and the role of the funders for the present study and, if applicable, for the original study on which the present article is based  **[Funding section]** |

*Give information separately for cases and controls in case-control studies and, if applicable, for exposed and unexposed groups in cohort and cross-sectional studies.

**Note:** An Explanation and Elaboration article discusses each checklist item and gives methodological background and published examples of transparent reporting. The STROBE checklist is best used in conjunction with this article (freely available on the Web sites of PLoS Medicine at http://www.plosmedicine.org/, Annals of Internal Medicine at http://www.annals.org/, and Epidemiology at http://www.epidem.com/). Information on the STROBE Initiative is available at www.strobe-statement.org.
